# Reflectance confocal microscopy and line-field confocal optical coherence tomography in hair loss: A systematic review and a glossary of nomenclature

**DOI:** 10.1016/j.jdin.2026.09.014

**Published:** 2026-10-06

**Authors:** Jakub Żółkiewicz, Marco Spadafora, Aleksandra Anderson, Marta Kurzeja, Roman J. Nowicki, Giovanni Pellacani, Lidia Rudnicka, Caterina Longo

**Affiliations:** a Department of Dermatology, Venereology and Allergology, Faculty of Medicine, Medical University of Gdańsk, Gdańsk, Poland; b Department and Clinic of Dermatology, Venereology and Allergology, University Clinical Center, Gdańsk, Poland; c Department of Dermatology, University of Modena and Reggio Emilia, Modena, Italy; d Department of Dermatology, Medical University of Warsaw, Warsaw, Poland; e Department of Clinical Sciences, Sapienza University of Rome, Rome, Italy

**Keywords:** cicatricial alopecia, hair loss, line-field confocal optical coherence tomography, noncicatricial alopecia, reflectance confocal microscopy, treatment monitoring

## Abstract

**Background:**

Reflectance confocal microscopy (RCM) and line-field confocal optical coherence tomography (LC-OCT) are emerging noninvasive imaging techniques in hair loss, potentially bridging the diagnostic gap between trichoscopy and histopathological examination.

**Objective:**

To systematically summarize RCM and LC-OCT features across cicatricial and noncicatricial alopecias.

**Methods:**

This systematic review was conducted according to the Preferred Reporting Items for Systematic Reviews and Meta-Analyses 2020 guidelines and was registered in the International Prospective Register of Systematic Reviews (CRD420251134481). MEDLINE and Embase were searched from inception to February 28, 2026. Eligible studies included original studies and case reports of RCM and/or LC-OCT in hair loss.

**Results:**

Forty-one studies encompassing 517 patients were included in the qualitative synthesis. Cicatricial alopecias showed higher pooled prevalence of dilated vessels, exocytosis, adnexal inflammatory infiltrates, and periadnexal sclerosis, whereas rimmed dermal papillae and hair shaft miniaturization predominated in noncicatricial forms. Evidence for treatment monitoring was limited but promising. A harmonized glossary linking RCM and LC-OCT features to trichoscopic and histopathological correlates was proposed.

**Limitations:**

This review is limited by the relatively low number of included studies and lack of standardized reporting of imaging features.

**Conclusion:**

RCM and LC-OCT are valuable adjunctive tools in hair loss diagnostics and may aid differentiation between cicatricial and noncicatricial forms.

**Capsule Summary**

• This systematic review synthesizes heterogeneously reported reflectance confocal microscopy and line-field confocal optical coherence tomography features of cicatricial and noncicatricial alopecias into the first quantitative synthesis with a unified glossary of nomenclature.
• Reflectance confocal microscopy and line-field confocal optical coherence tomography may serve as adjunct tools to trichoscopy and histopathology in differential diagnosis of alopecias and to monitor treatment response.

## Introduction

Hair loss encompasses a heterogeneous spectrum of cicatricial and noncicatricial disorders associated with substantial clinical and psychosocial burden. Accurate and early identification of the underlying cause is essential for appropriate management, as delayed diagnosis may lead to permanent hair loss.^1^

Over the past 2 decades, trichoscopy has become an integral component of the diagnostic evaluation of hair loss, complementing clinical assessment and histopathological examination. It has improved diagnostic accuracy and, in selected subtypes, allows early assessment of therapeutic response.^2^ Nevertheless, trichoscopy may be insufficient to identify the cause of hair loss, particularly in cicatricial or early stages. In such cases, scalp biopsy with histopathological examination remains essential, although it is invasive and is impractical for longitudinal treatment monitoring.

Reflectance confocal microscopy (RCM) and line-field confocal optical coherence tomography (LC-OCT) are increasingly used to investigate hair loss. Both provide dynamic, noninvasive visualization of skin and follicles at near-histological resolution and may help select biopsy sites, distinguish active inflammation from established scarring, and detect hair regrowth before clinical change.

This systematic review synthesized RCM and LC-OCT features across alopecias, evaluated diagnostic and treatment-monitoring applications, and proposed a harmonized glossary of nomenclature.

## Materials and methods

This systematic review was conducted in accordance with the Preferred Reporting Items for Systematic Reviews and Meta-Analyses 2020 guidelines.^3^ The study protocol was prospectively registered in the International Prospective Register of Systematic Reviews (CRD420251134481).

Eligible studies included original research articles, case series, and case reports describing RCM and/or LC-OCT features in patients with hair loss without age limitation. Treatment-monitoring studies were also included. Review articles, conference abstracts, editorials, publications lacking imaging data, or with concomitant scalp disorders potentially affecting imaging interpretation were excluded.

MEDLINE and Embase were searched from inception to February 28, 2026, alongside forward and backward citation chaining in Web of Science. No language restrictions were applied. Retrieved records were deduplicated and screened independently by 2 reviewers (J.Ż. and M.S.) against predefined criteria. Full texts were evaluated independently by the same reviewers, with discrepancies resolved by a third reviewer (C.L.). Data extraction was performed by a single reviewer (J.Ż.) using a predefined form and verified by a second reviewer (C.L.). Extracted data included study design, alopecia subtype, imaging modality, and all reported RCM and/or LC-OCT features. Denominators indicate individual patients, with 1 observation per assessed feature. Unreported variables were classified as unassessed. Among publications concerning the same disease, potential cohort overlap was assessed by comparing author groups, recruiting centers, and reported recruitment periods.

Risk of bias was assessed independently by 2 reviewers using Joanna Briggs Institute Critical Appraisal Checklists^4^, ^5^ appropriate to each study design. Studies were classified as low risk if more than 70% of applicable criteria were fulfilled, moderate risk if more than 50%, and high risk otherwise (Table I). Levels of evidence were assigned according to the Oxford 2011 classification.^47^ The certainty of evidence for each outcome of the subgroup analysis was assessed using the Grading of Recommendations, Assessment, Development and Evaluation approach.^48^ Results were synthesized narratively. The quantitative synthesis was restricted to pooled prevalence, as the primary studies lacked formal diagnostic-accuracy designs. For each RCM feature, pooled prevalence was estimated using random-effects meta-analysis with logit transformation, computed in R version 4.5.2 with the meta package (metaprop function). Between-study variance was estimated by restricted maximum likelihood, with a continuity correction of 0.5 applied to all cohorts. Subgroup analyses compared cicatricial and noncicatricial alopecias. Publications reporting several cicatricial entities jointly were entered as single mixed cicatricial cohorts. Differences in pooled prevalence were tested with the χ^2^ test under the random-effects model. Heterogeneity was assessed using the I^2^ statistic (<25%, low; 25% to 50%, moderate; >50% to 75%, substantial; >75%, high). Pooled analyses were not performed for LC-OCT features due to limited data. Three post hoc sensitivity analyses were performed (Supplementary File 8, available via Mendeley at https://data.mendeley.com/datasets/y9m58jsn7d/4). Provided figures did not contribute to the evidence synthesis, to the quantitative analysis, or to the conclusions of this review. The complete search strategy and Joanna Briggs Institute Critical Appraisal Checklists are available via Mendeley at https://data.mendeley.com/datasets/y9m58jsn7d/4.^49^

**Table I:** Risk of bias assessment

Author	Year	JBI tool used	Q1	Q2	Q3	Q4	Q5	Q6	Q7	Q8	Q9	Q10	Criteria fulfilled, *n*/*N* (%)	Risk of bias	Oxford level of evidence
Agozzino^6^	2011	CRS	**Yes**	**Yes**	**Yes**	*Unclear*	*Unclear*	**Yes**	**Yes**	**Yes**	**Yes**	**Yes**	8/10 (80%)	Low	5
Agozzino^7^	2015	CR	**Yes**	**Yes**	**Yes**	**Yes**	**Yes**	**Yes**	**Yes**	**Yes**	-	-	8/8 (100%)	Low	5
Annunziata^8^	2023	CSS	**Yes**	**Yes**	**Yes**	**Yes**	*Unclear*	*Unclear*	**Yes**	**Yes**	-	-	6/8 (75%)	Low	3
Ardigò^9^	2011	CRS	**Yes**	**Yes**	**Yes**	*Unclear*	*Unclear*	**Yes**	**Yes**	**Yes**	**Yes**	**Yes**	8/10 (80%)	Low	5
Ardigò^10^	2014	CR	**Yes**	**Yes**	**Yes**	**Yes**	**Yes**	**Yes**	**Yes**	**Yes**	-	-	8/8 (100%)	Low	5
Ardigò^11^	2016	CSS	**Yes**	**Yes**	**Yes**	**Yes**	**Yes**	**Yes**	No	**Yes**	-	-	7/8 (88%)	Low	3
Cantelli^12^	2020	CRS	**Yes**	**Yes**	**Yes**	*Unclear*	*Unclear*	**Yes**	**Yes**	**Yes**	**Yes**	*NA*	7/9 (78%)	Low	5
Castañeda-Yépiz^13^	2018	CR	**Yes**	*Unclear*	**Yes**	*Unclear*	*Unclear*	No	No	**Yes**	-	-	3/8 (38%)	High	5
Cinotti^14^	2015	CRS	**Yes**	**Yes**	**Yes**	*Unclear*	*Unclear*	**Yes**	**Yes**	*NA*	**Yes**	*NA*	6/8 (75%)	Low	5
Ciobotariu^15^	2025	CRS	**Yes**	**Yes**	**Yes**	**Yes**	**Yes**	**Yes**	**Yes**	No	**Yes**	**Yes**	9/10 (90%)	Low	5
Falcinelli^16^	2023	CR	**Yes**	**Yes**	**Yes**	**Yes**	**Yes**	**Yes**	No	**Yes**	-	-	7/8 (88%)	Low	5
Franceschini^17^	2019	CSS	**Yes**	**Yes**	**Yes**	**Yes**	**Yes**	No	**Yes**	**Yes**	-	-	7/8 (88%)	Low	3
Gori^18^	2023	CR	**Yes**	**Yes**	**Yes**	**Yes**	**Yes**	**Yes**	**Yes**	**Yes**	-	-	8/8 (100%)	Low	5
Khan^19^	2023	CR	**Yes**	**Yes**	**Yes**	**Yes**	**Yes**	**Yes**	No	**Yes**	-	-	7/8 (88%)	Low	5
Kurzeja^20^	2021	CRS	**Yes**	**Yes**	**Yes**	*Unclear*	*Unclear*	**Yes**	**Yes**	No	**Yes**	**Yes**	7/10 (70%)	Moderate	5
Kurzeja^21^	2023	CRS	**Yes**	**Yes**	**Yes**	*Unclear*	*Unclear*	**Yes**	**Yes**	No	**Yes**	*NA*	6/9 (67%)	Moderate	5
Kurzeja^22^	2024	CRS	**Yes**	**Yes**	**Yes**	*Unclear*	*Unclear*	**Yes**	**Yes**	**Yes**	**Yes**	**Yes**	8/10 (80%)	Low	5
Lacarrubba^23^	2024	CSS	No	**Yes**	**Yes**	**Yes**	No	No	**Yes**	**Yes**	-	-	5/8 (63%)	Moderate	3
Mandel^24^	2019	CR	**Yes**	**Yes**	**Yes**	**Yes**	**Yes**	**Yes**	No	**Yes**	-	-	7/8 (88%)	Low	5
Mazzilli^25^	2020	CSS	**Yes**	**Yes**	**Yes**	**Yes**	No	No	**Yes**	**Yes**	-	-	6/8 (75%)	Low	3
Mazzilli^26^	2020	CR	**Yes**	**Yes**	**Yes**	**Yes**	**Yes**	No	No	**Yes**	-	-	6/8 (75%)	Low	5
Megna^27^	2023	CR	**Yes**	**Yes**	**Yes**	**Yes**	**Yes**	**Yes**	**Yes**	**Yes**	-	-	8/8 (100%)	Low	5
Melo^28^	2020	CSS	**Yes**	**Yes**	**Yes**	**Yes**	**Yes**	**Yes**	**Yes**	**Yes**	-	-	8/8 (100%)	Low	3
Napolitano^29^	2022	CSS	**Yes**	**Yes**	**Yes**	**Yes**	**Yes**	No	**Yes**	No	-	-	6/8 (75%)	Low	5
Nutz^30^	2024	CSS	**Yes**	**Yes**	**Yes**	**Yes**	**Yes**	No	**Yes**	**Yes**	-	-	7/8 (88%)	Low	3
Nutz^31^	2025	CSS	**Yes**	**Yes**	**Yes**	**Yes**	**Yes**	No	**Yes**	**Yes**	-	-	7/8 (88%)	Low	3
Peccerillo^32^	2020	CR	**Yes**	**Yes**	**Yes**	**Yes**	**Yes**	**Yes**	**Yes**	**Yes**	-	-	8/8 (100%)	Low	5
Piscazzi^33^	2024	CSS	**Yes**	**Yes**	**Yes**	**Yes**	No	No	**Yes**	**Yes**	-	-	6/8 (75%)	Low	3
Rubin^34^	2023	CRS	**Yes**	**Yes**	**Yes**	*Unclear*	*Unclear*	**Yes**	**Yes**	**Yes**	**Yes**	*NA*	7/9 (78%)	Low	5
Rudnicka^35^	2008	CR	No	No	**Yes**	**Yes**	**Yes**	No	No	**Yes**	-	-	4/8 (50%)	High	5
Starace^36^	2021	CSS	**Yes**	**Yes**	**Yes**	**Yes**	No	No	No	*NA*	-	-	4/7 (57%)	Moderate	5
Starace^37^	2024	CSS	**Yes**	**Yes**	**Yes**	**Yes**	No	No	**Yes**	**Yes**	-	-	6/8 (75%)	Low	3
Starace^38^	2025	CSS	**Yes**	**Yes**	**Yes**	**Yes**	**Yes**	*Unclear*	**Yes**	**Yes**	-	-	7/8 (88%)	Low	3
Thompson^39^	2021	CRS	**Yes**	**Yes**	**Yes**	*Unclear*	*Unclear*	*Unclear*	**Yes**	No	*Unclear*	*NA*	4/9 (44%)	High	5
Thompson^40^	2022	CR	No	No	**Yes**	**Yes**	**Yes**	*Unclear*	No	**Yes**	-	-	4/8 (50%)	High	5
Tognetti^41^	2024	CRS	**Yes**	**Yes**	**Yes**	*Unclear*	*Unclear*	**Yes**	**Yes**	**Yes**	**Yes**	*NA*	7/9 (78%)	Low	5
Traini^42^	2026	CSS	**Yes**	**Yes**	**Yes**	**Yes**	No	No	**Yes**	**Yes**	-	-	6/8 (75%)	Low	3
Trovato^43^	2025	CSS	*Unclear*	**Yes**	**Yes**	**Yes**	*Unclear*	*Unclear*	No	*NA*	-	-	3/7 (43%)	High	5
Veasey^44^	2019	CR	**Yes**	No	**Yes**	**Yes**	**Yes**	No	No	**Yes**	-	-	5/8 (63%)	Moderate	5
Verzì^45^	2024	CSS	**Yes**	**Yes**	**Yes**	**Yes**	*Unclear*	*Unclear*	**Yes**	*NA*	-	-	5/7 (71%)	Low	5
Wang^46^	2023	CRS	**Yes**	**Yes**	**Yes**	*Unclear*	*Unclear*	**Yes**	**Yes**	**Yes**	**Yes**	*NA*	7/9 (78%)	Low	5

Results are reported as the number of applicable criteria fulfilled out of the number of applicable criteria, with the percentage in parentheses. Items rated “not applicable” are excluded from the denominator, which, therefore, varies with checklist length. An “unclear” response indicates that the available information was insufficient to establish that the criterion had been fulfilled. It was retained as “unclear” in the item-level assessment and did not contribute to the numerator. Because the JBI tools do not prescribe thresholds for overall risk-of-bias categories, we followed cutoffs used in previous studies: low risk when >70% of applicable criteria were fulfilled, moderate risk when >50%, and high risk otherwise. These categories were descriptive and did not affect study inclusion or weighting.

*CR*, Case report; *CRS*, case report series; *CSS*, cross-sectional study; *JBI*, Joanna Briggs Institute; *NA*, not applicable.

## Results

### Study selection and characteristics

The database search identified 1978 records, and citation chaining identified a further 737. After removal of duplicates, 2439 unique titles and abstracts were screened and 68 full-text articles were assessed for eligibility. Ultimately, 41 studies (16 original studies, 12 case series, and 13 case reports) encompassing 517 patients met the inclusion criteria (Fig 1).

**Fig 1:**
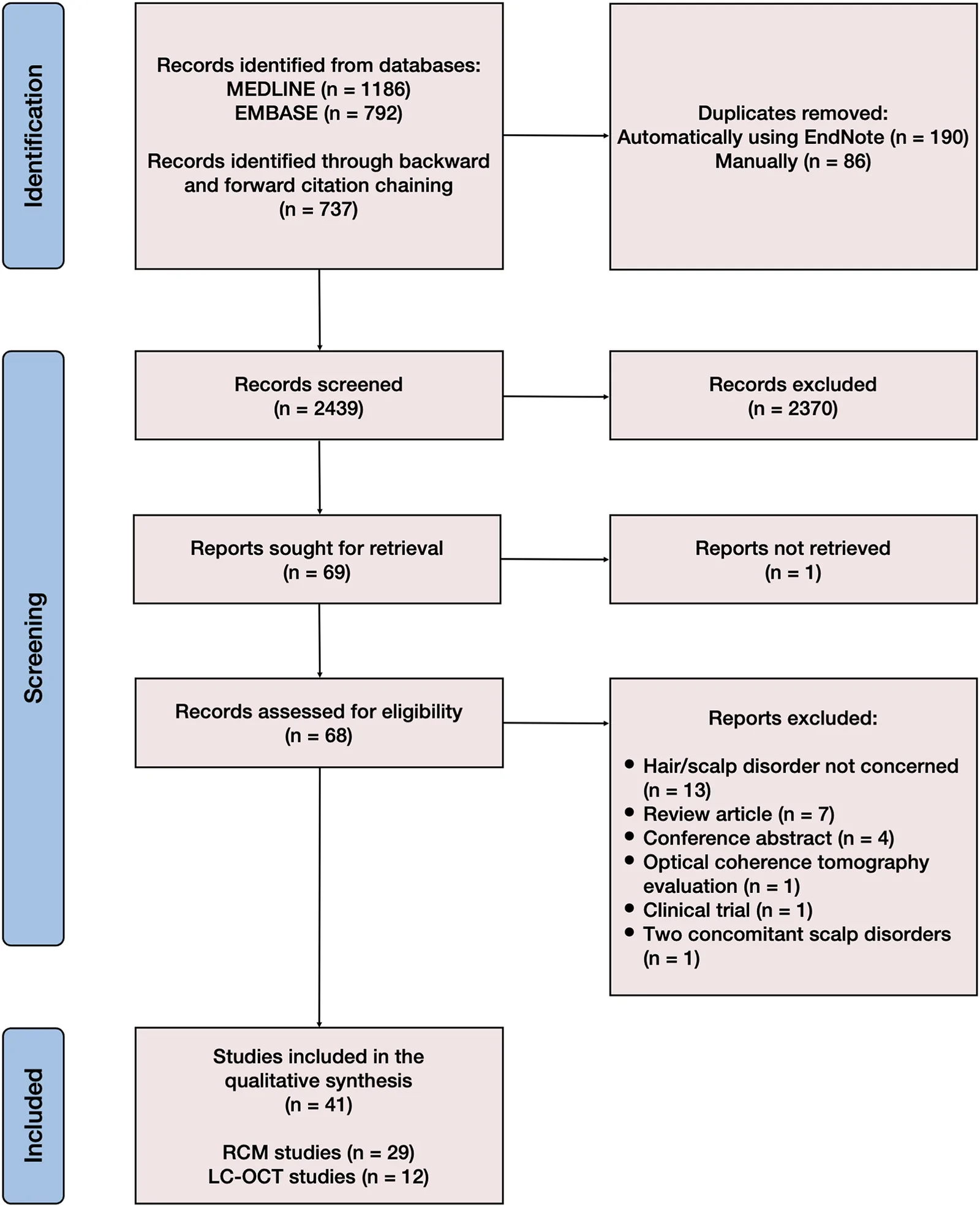
PRISMA flow diagram of study identification and selection. *PRISMA*, Preferred Reporting Items for Systematic Reviews and Meta-Analyses.

Noncicatricial alopecias were investigated in 21 studies (15 RCM and 6 LC-OCT), cicatricial alopecias in 17 studies (12 RCM and 5 LC-OCT), and other subtypes in 7 studies (6 RCM and 1 LC-OCT). Trichoscopy data were provided in 30 studies, and treatment monitoring was reported in 10 RCM and 2 LC-OCT studies. Overall, 31 studies demonstrated low risk of bias, 5 moderate, and 5 high (Table I). No definite cohort overlap was identified. However, overlap could not be excluded between the alopecia areata (AA) cohorts reported by Ardigò et al^11^ and Ardigò et al^9^, as neither study provided recruitment dates. Table II, Table III, Table IV, Table V summarize the most frequently reported features and harmonized terminology. Complete extraction and evidence tables are available via Mendeley at https://data.mendeley.com/datasets/y9m58jsn7d/4.^49^

**Fig 2:**
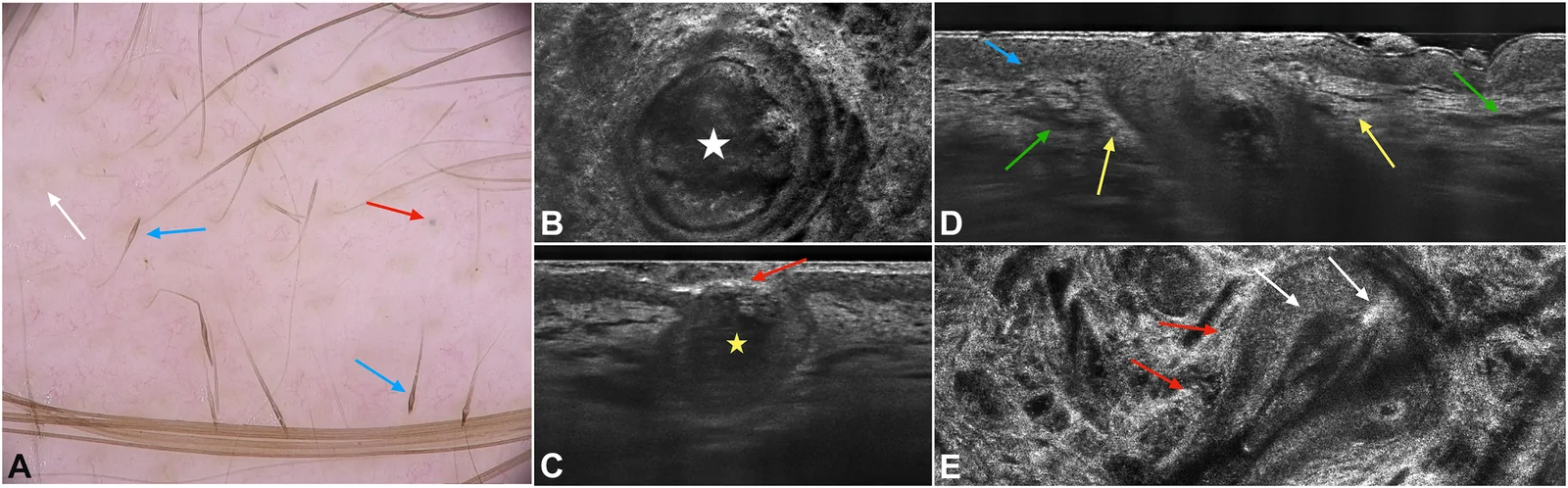
Alopecia areata (AA)–trichoscopic and line-field confocal optical coherence tomography (LC-OCT) presentation. **A,** On trichoscopy, exclamation mark hairs (*blue arrows*), *yellow dots* (*white arrow*), and *black dots* (*red arrow*) are identified. **B,** At the site corresponding to the trichoscopic *yellow dot*, LC-OCT reveals hyper-reflective amorphous material filling a dilated hair infundibulum (*white asterisk*). **C,** In the same lesion and corresponding to panel **(B)**, vertical LC-OCT plane demonstrates hyper-reflective amorphous material within the dilated infundibulum (*red arrow*), overlying a hyporeflective roundish area (*yellow asterisk*). **D,** Vertical planes reveal dermal inflammatory infiltrates (*yellow arrows*), elongated blood vessels (*green arrows*), and reduced undulation of the dermo-epidermal junction (*blue arrow*). **E,** In horizontal planes, small hyper-reflective cells corresponding to peri-infundibular (*red arrows*) and infundibular (*white arrows*) inflammatory infiltrates are observed. **A,** FotoFinder Medicam 1000; FotoFinder Systems GmbH, Bad Birnbach, Germany; 20× magnification; immersion: ultrasound gel; **B-E,** deepLive; DAMAE Medical, Paris, France.

**Fig 3:**
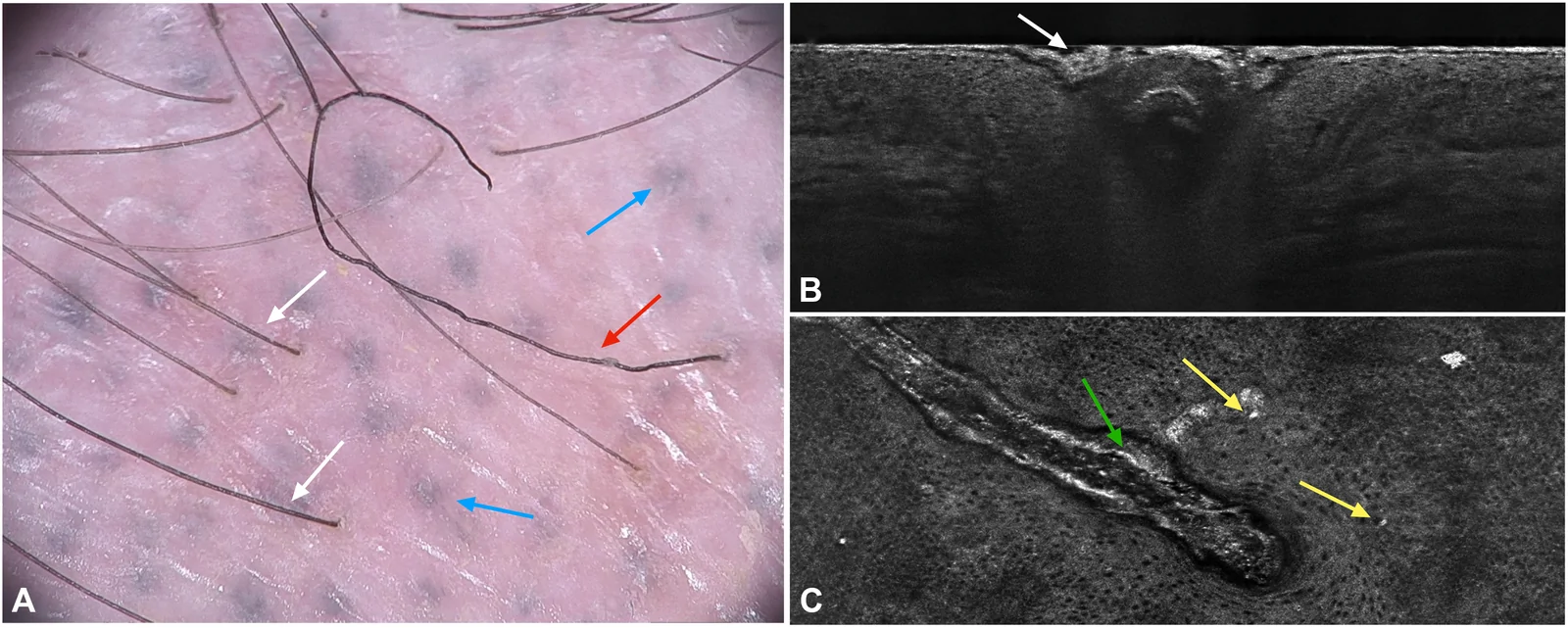
Frontal fibrosing alopecia (FFA)–trichoscopic and line-field confocal optical coherence tomography (LC-OCT) presentation. **A,** Trichoscopy of FFA reveals “lonely hair” sign (*white arrows*), pili torti (*red arrow*) overlying milky-red structureless areas with visible *black dots* (*blue arrows*) due to intentional micropigmentation. **B,** LC-OCT vertical planes reveal a single hair in the hair unit surrounded by infundibular hyperkeratosis corresponding to peripilar cast on trichoscopy (*white arrow*). **C,** LC-OCT horizontal planes show degeneration of the hair shaft morphology corresponding to pili torti (*green arrow*) on trichoscopy and perifollicular inflammatory infiltrates (*yellow arrows*). **A,** FotoFinder Medicam 1000; FotoFinder Systems GmbH, Bad Birnbach, Germany; 20× magnification; no immersion; **B** and **C,** deepLive; DAMAE Medical, Paris, France.

**Fig 4:**
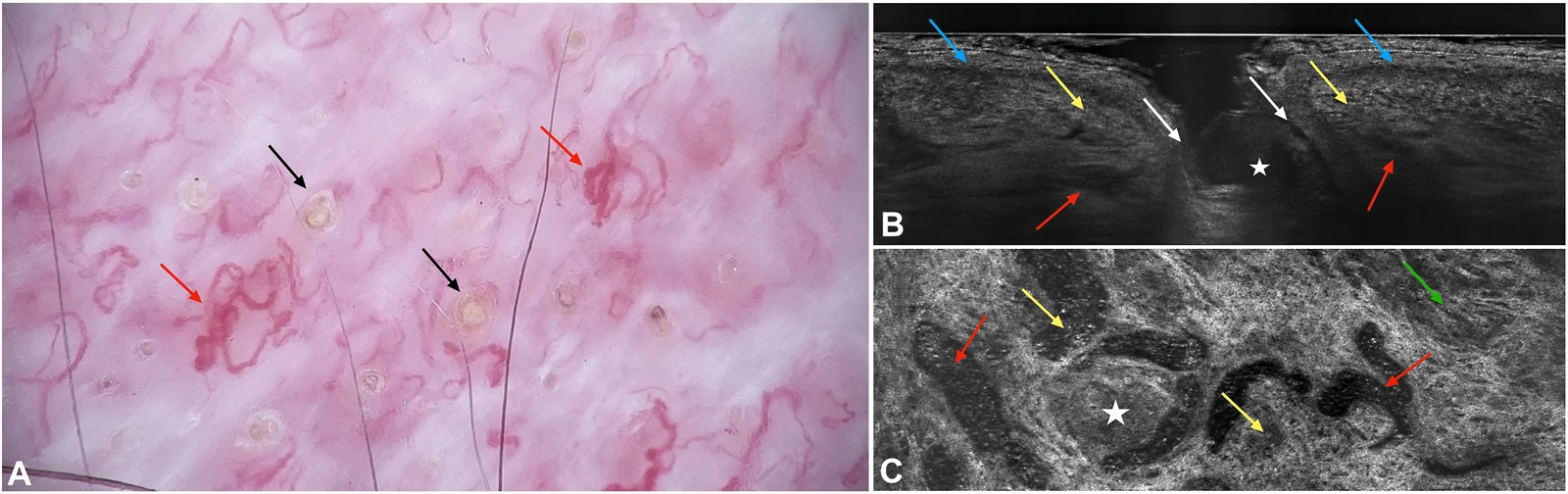
Discoid lupus erythematosus (DLE)–trichoscopic and line-field confocal optical coherence tomography (LC-OCT) presentation. **A,** Trichoscopy of DLE of the scalp reveals *yellow dots* (*black arrows*) and thick branching vessels (*red arrows*) overlying milky-red structureless areas. **B,** LC-OCT vertical planes reveal blurring of the dermo-epidermal junction (*blue arrows*) secondary to interface dermatitis (*yellow arrows*), dilated blood vessels (*red arrows*), and mucin deposits (*white arrows*) next to follicular remnants (*white asterisk*). **C,** LC-OCT horizontal planes show follicular remnants (*white asterisk*), dilated blood vessels (*red arrows*), dermal inflammatory infiltrates (*yellow arrows*), and hyper-reflective collagen bundles corresponding to dermal sclerosis (*green arrow*). **A,** FotoFinder Medicam 1000; FotoFinder Systems GmbH, Bad Birnbach, Germany; 40× magnification; immersion: ultrasound gel; **B** and **C,** deepLive; DAMAE Medical, Paris, France.

**Fig 5:**
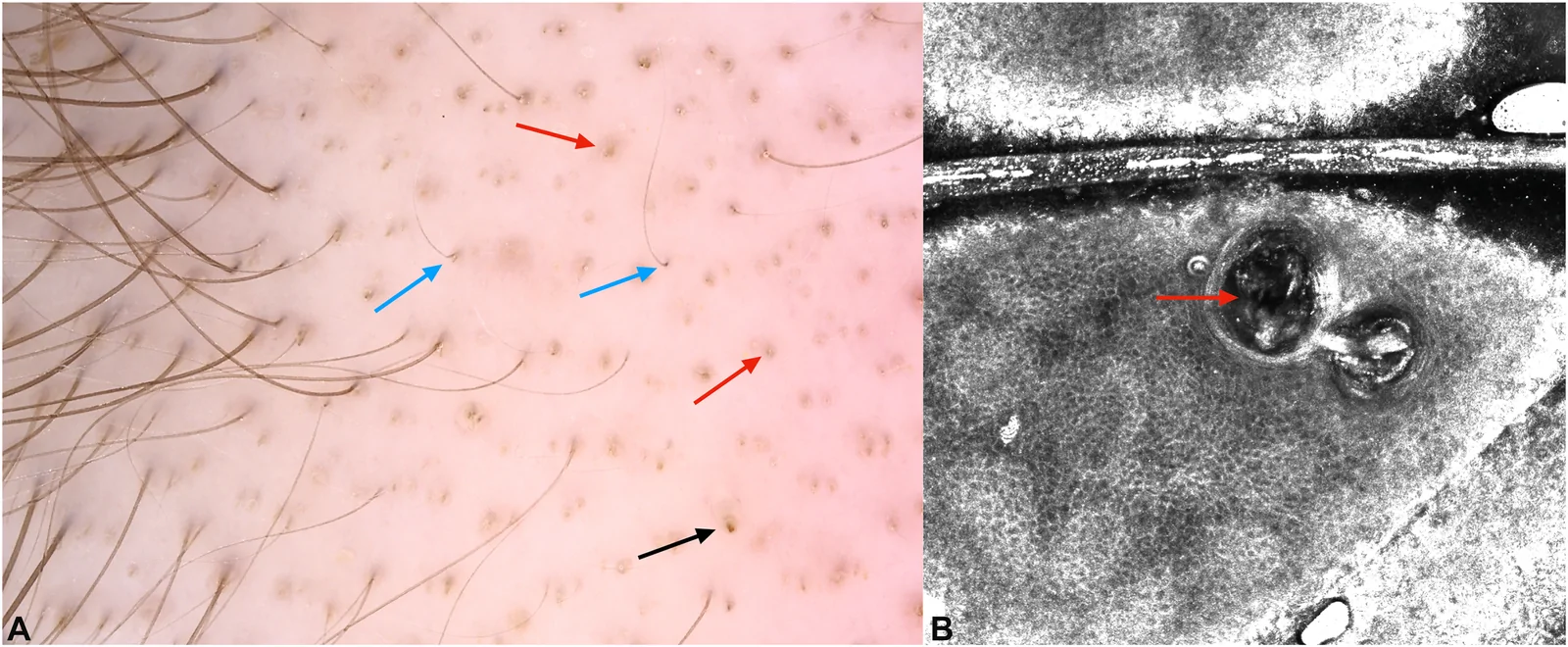
Alopecia areata (AA)–trichoscopic and reflectance confocal microscopy (RCM) presentation. **A,** Trichoscopy in a patient with AA treated with upadacitinib for 8 weeks demonstrates *yellow dots* (*red arrows*), *black dots* (*black arrow*), and upright regrowing hairs (*blue arrows*). **B,** RCM reveals hyper-reflective amorphous material filling the upper segment of a dilated hair follicle (*red arrow*), corresponding to the *yellow dots* on trichoscopy. **A,** Canfield D200 EVO; Canfield Scientific GmbH, Bielefeld, Germany; 15× magnification; immersion: ultrasound gel; **B,** VivaScope 1500; Caliber Imaging and Diagnostics, Rochester, NY, USA.

**Fig 6:**
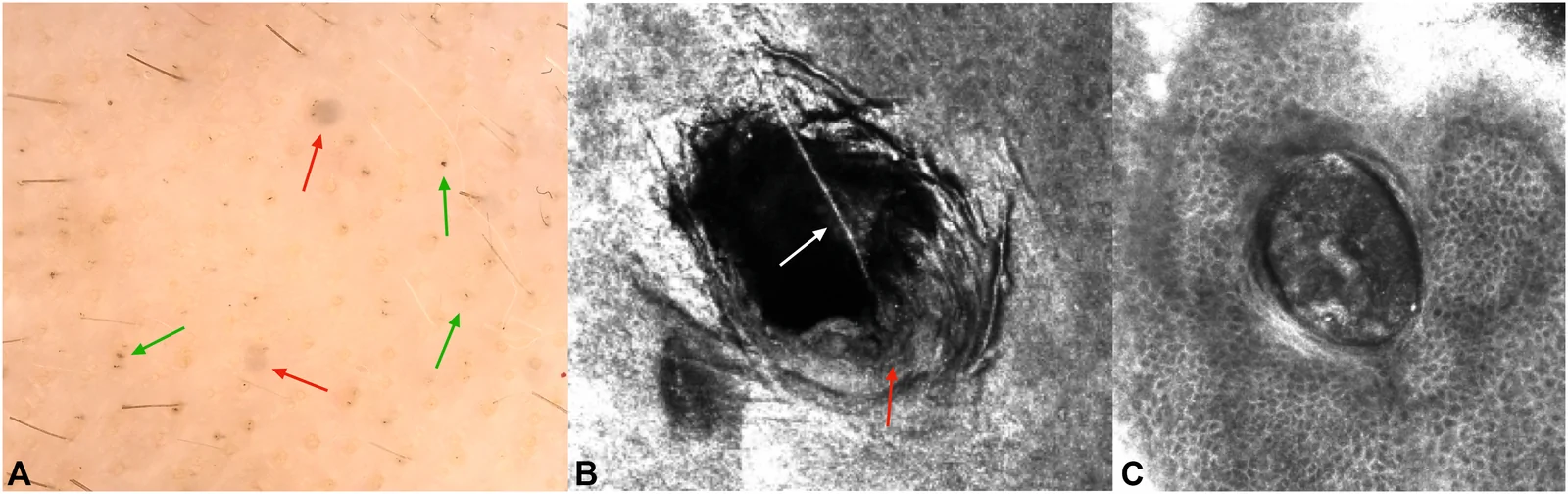
Chemotherapy-induced alopecia (CIA)–trichoscopic and reflectance confocal microscopy (RCM) presentation. **A,** Trichoscopy reveals numerous *black dots* (*green arrows*) and CIA *dots* (*red arrows*). **B,** On RCM, CIA *dots* correspond to follicular structures containing a residual hair shaft remnant (*white arrow*) surrounded by necrotic material (*red arrow*). **C,***Black dots* are visible on RCM as normal-sized hair follicles filled with homogeneous, highly reflective material. **A,** Canfield D200 EVO; Canfield Scientific GmbH, Bielefeld, Germany; 15× magnification; immersion: ultrasound gel; **B** and **C,** VivaScope 1500; Caliber Imaging and Diagnostics, Rochester, NY, USA.

**Table II:** Most frequently reported reflectance confocal microscopy features according to the diagnosis∗

Entity	RCM feature	Frequency
Noncicatricial alopecias		
Alopecia areata Alopecia areata incognita†	Follicles in telogen†	14/14 (100%)
	Catagen and telogen germinal units†	13/14 (92.9%)
	Rimmed dermal papillae	15/19 (78.9%)
	Diffuse dermal fibers degeneration	26/34 (76.5%)
	Hair shaft miniaturization	24/33 (72.7%)
	Yellow dots/empty infundibular ostia	24/35 (68.6%)
	Exocytosis	11/20 (55%)
Androgenetic alopecia	Rimmed dermal papillae	29/35 (82.9%)
	Hair shaft miniaturization	26/36 (72.2%)
	Dermal fibrosis	18/35 (51.4%)
Chemotherapy-induced alopecia	Abnormal follicular morphology	22/24 (91.7%)
	CIA dots	71/87 (81.6%)
	Black dots	82/111 (73.9%)
	Yellow dots	78/111 (70.3%)
	Hair shaft miniaturization	69/101 (68.3%)
	Abnormal hair shaft morphology	64/111 (57.7%)
Cicatricial alopecias		
Discoid lupus erythematosus	Epidermal disarray	14/14 (100%)
	Necrotic keratinocytes	12/12 (100%)
	Exocytosis	12/14 (85.7%)
	Hyperkeratotic infundibulum	10/12 (83.3%)
	Interface changes	11/15 (73.3%)
	Dilated vessels	10/15 (66.7%)
	Dermal sclerosis/abnormal collagen bundles	10/15 (66.7%)
	Dilated infundibulum	8/12 (66.7%)
	Spongiosis	9/14 (64.3%)
	Adnexal inflammatory infiltrates	9/15 (60%)
Folliculitis decalvans	Dilated vessels	25/25 (100%)
	Exocytosis	15/15 (100%)
	Obscuration of the dermal papillae	23/25 (92%)
	Dermal fibrosis/scarring	23/25 (92%)
	Perifollicular fibrosis	22/24 (91.7%)
	Hair tufts	30/39 (76.9%)
	Dermal fibrosis/scarring–interfollicular	18/24 (75%)
	Perifollicular inflammatory infiltrates	11/15 (73.3%)
	Follicular pustule	25/39 (64.1%)
	Hyper/parakeratosis	7/14 (50%)
Lichen planopilaris	Dermal inflammatory infiltrates	5/5 (100%)
	Dermal and/or perifollicular sclerosis	5/5 (100%)
	Adnexal inflammatory infiltrates	5/5 (100%)
	Exocytosis	5/5 (100%)

*CIA*, Chemotherapy-induced alopecia; *RCM*, reflectance confocal microscopy.

∗ Only features reported in at least 5 cases and present in ≥50% of patients are listed according to frequency.

† Features described in the alopecia areata incognita.

**Table III:** Most frequently reported line-field confocal optical coherence tomography features according to the diagnosis∗

Entity	LC-OCT feature	Frequency
Noncicatricial alopecias		
Alopecia areata	Reduced undulation of the DEJ profile	20/20 (100%)
	Yellow-white dots	63/85 (74.1%)
	Follicular remnants/black dots	59/96 (61.5%)
Cicatricial alopecias		
Discoid lupus erythematosus	Perifollicular fibrosis	4/4 (100%)
	Periadnexal inflammatory infiltrates	4/4 (100%)
	Interface changes	18/19 (94.7%)
	Dermal sclerosis/abnormal collagen bundles	18/19 (94.7%)
	No rimming of the papillae	14/15 (93.3%)
	Basal cell vacuolization	17/19 (89.5%)
	Mucin deposits	15/17 (88.2%)
	Melanophages	9/15 (60%)
	Follicular remnants	13/19 (68.4%)
Folliculitis decalvans	Hair tufts	8/9 (88.9%)
	Dilated vessels	4/5 (80%)
	Dermal and perifollicular scarring	7/9 (77.8%)
	Perifollicular inflammatory infiltrates	7/9 (77.8%)
	Perifollicular pustule	6/9 (66.7%)
Frontal fibrosing alopecia†	Dermal sclerosis	29/29 (100%)
	Interface changes	5/5 (100%)
	Perifollicular fibrosis	5/5 (100%)
	Perifollicular inflammatory infiltrates	23/24 (95.8%)
	Infundibular hyperkeratosis	19/24 (79.2%)
	Hypervascularization	19/24 (79.2%)
	Dilated vessels	15/24 (62.5%)
Lichen planopilaris†	Interface changes	10/10 (100%)
	Dermal and/or perifollicular sclerosis	37/38 (97.4%)
	Perifollicular inflammatory infiltrates	24/28 (85.7%)
	Hypervascularization	19/26 (73.1%)
	Infundibular hyperkeratosis	16/27 (59.3%)
	Adnexal inflammatory infiltrates	5/5 (100%)
	Exocytosis	5/5 (100%)

*DEJ*, Dermo-epidermal junction; *LC-OCT*, line-field confocal optical coherence tomography.

∗ Only features reported in at least 5 cases and present in ≥50% of patients are listed according to frequency.

† Features identified in the transitional zone.

**Table IV:** Glossary of reflectance confocal microscopy features in hair loss

RCM term	Definition	Histopathologic correlate	Trichoscopy correlate	Entities	Source (reference)
Epidermal features					
Exocytosis	Single, aggregates or diffuse round-to-polygonal, mildly refractive cells at the level of stratum spinosum	Exocytosis	-	AA, AGA, CIA, DLE, FD, FFA, LPP, fMF, LSC	6, 7, 10, 11, 17, 20, 25, 28, 29, 34, 36, 38
Spongiosis	A darker area relative to the surrounding epithelium of the stratum spinosum with intercellular spaces between keratinocytes larger than normal	Spongiosis	-	DLE, FD, FFA, LPP, LSC	6, 28, 32, 36
Epidermal disarray	Partial or complete loss of the normal honeycomb-like architecture of the epidermis at the level of the stratum spinosum	Epidermal disarray	-	DLE, FFA, LPP	6, 28
Necrotic keratinocytes	Pale, ill-defined, round-to-polygonal cells, larger than normal keratinocytes, at the level of the stratum spinosum	Necrotic keratinocytes	-	DLE	^28^
Pigment incontinence	White, differently shaped inclusions between the epidermal cells	Pigment incontinence	-	FFA, LPP	^20^
Hyperkeratosis	Bright amorphous material with detached keratinocytes in the stratum corneum	Hyperkeratosis	Scales	FD, LSC	25, 36
Parakeratosis	Highly refractile, polygonal structures at the level of the stratum corneum, similar to or smaller in size than normal keratinocytes (corresponding to the remains of the keratinocyte nuclei)	Parakeratosis	Scales	FD, fMF, PsA, LSC	10, 25, 27, 36
Hyphae in the stratum corneum/epidermis	Sparse elongated, branched structures with high reflectivity and/or speckled appearance in the stratum corneum/epidermis	Hyphae in the stratum corneum/epidermis	-	Tinea capitis, kerion celsi	14, 19, 24, 44
DEJ-associated features					
Rimmed dermal papillae	Well-demarcated rings comprised of bright cells around a dark center	Integrity and pigmentation of the dermo-epidermal junction	Honeycomb pattern (hyperchromic grid with irregular lines and holes suggesting sun-damaged skin)	AA, AGA, CIA, FD	11, 33, 34, 38
Interface changes	Round-to-polygonal, mildly refractive cells at the level of the DEJ, as singles or clusters, associated with total or partial obliteration of the ring-like structures around the dermal papillae	Interface changes	-	DLE, FFA, LPP	6, 28
Obscuration of dermal papillae	Total or partial absence of papillary rings	-	-	FD, ECDS	32, 46
Adnexal and periadnexal features					
Yellow dots/infundibular hyperkeratosis/follicular keratinization	Bright material filling the (dilated) follicular ostia	Infundibular hyperkeratosis/Hair follicle filled with keratotic material and/or sebum	Yellow dots	AA, AGA, CIA, HHPi-induced alopecia, DLE, FFA, LPP, LSC	8, 9, 11, 12, 17, 20, 28, 29, 34, 36, 37, 38
Adnexal inflammatory infiltrates	Single, aggregates or diffuse round-to-polygonal, mildly refractive cells located in the adnexal structures	Infiltration of inflammatory cells in adnexal structures	-	AA, AGA, CIA, DLE, FD, FFA, LPP, fMF, LSC	6, 7, 10, 11, 28, 32, 36, 38
Periadnexal/perifollicular inflammatory infiltrates	Single, aggregates or diffuse round-to-polygonal, mildly refractive cells located around the hair follicles	Periadnexal inflammatory cells	-	AA incognita, FD, FFA, LPP	20, 25, 37
Periadnexal/perifollicular sclerosis/fibrosis/scarring/dermal fibre degeneration	Increased number of white, ill-defined, coarse dermal fibers around adnexal structures/periadnexal dermal fibre degeneration	Periadnexal sclerosis/abnormal collagen bundles/scarring	Perifollicular white ring	AA, AGA, FD, FFA, LPP	11, 20, 33
Black dots/cadaverized hairs	Homogeneous and reflective material filling follicular ostia	Hair medulla remnants	Black dots	CIA	8, 17, 38
CIA dots/CIA-like dots	Mashed, malted, nonhomogeneous material inside the follicular ostia, may contain normal or fragmented hair	-	Yellow structureless area surrounded by a thin white collarette	CIA, HHPi-induced alopecia	12, 17, 38
Perifollicular hyperkeratosis/peripilar casts	Hyperkeratotic, white ring surrounding the follicular ostium	Perifollicular hyperkeratosis	Peripilar casts	FFA	28, 39, 40
Abnormal follicular morphology	Dystrophic aspect and/or high number of empty follicles	-	-	CIA	^8^
Dilated infundibulum	Black round or oval lumina, ranging in diameter from 140 μm to 190 μm (in comparison with normal adnexal infundibula ranging from 45 μm to 80 μm) in the dermis	Dilated infundibulum	-	DLE	^28^
Hair shaft emerging from distorted infundibula	Hair shaft emerging from distorted infundibula	-	-	FFA	^39^
Follicular pustule	Dome-shaped (round to oval) lesion with a well-defined black halo and granular, homogeneous or heterogeneous material inside	Intrafollicular pustule	Follicular pustule	FD	25, 32, 33
Hair shaft-related features					
Abnormal hair shaft morphology	Irregular diameter and length of hair shafts, “salt and pepper” hairs, hair shaft constrictions, broken appearance at the end of the hair shaft	-	-	CIA, Monilethrix	8, 13, 17, 38
Hair shaft thickness heterogeneity/miniaturization	Reduction of the caliber of the hair shaft (diameter <0.03 mm) in comparison to normal hair, reduction of the caliber of the adnexal infundibulum	Hair shaft miniaturization, miniaturized follicles	Hair shaft thickness heterogeneity/miniaturization	AA, AGA, CIA	8, 11, 34, 35, 37, 38
Ectothrix conidia	Roundish homogeneous (5–10 μm in size) hyper-reflective structures in the stratum corneum and/or on the surface of hair	Ectothrix conidia	-	Tinea capitis	^14^
Hair tufts	Dilated follicular opening with multiple hairs (>5 or 6 hair shafts) emerging from 1 ostium	Fibrous tissue contraction causing an epidermal depression and clustering of the inflamed follicular units that open into a common ostium	Hair tufts	FD	25, 32, 33
Catagen germinal units	Infundibular units connected to the hair shaft	Catagen germinal units	-	AA incognita	^37^
Telogen germinal units	Dilated infundibular unit associated to fibrotic tissue at the level of the superficial dermis	Telogen germinal units	-	AA incognita	^37^
Regrowing hairs	Regrowing hairs (eg circle hair, pigtail hair, and vellus hair)	-	Regrowing hairs (eg circle hair, pigtail hair, thin, short, not hypopigmented hair)	CIA	17, 38
Dermal features					
Dermal inflammatory infiltrates	Single, aggregates or diffuse round-to-polygonal, mildly refractive cells located in the dermis	Dermal inflammatory cells	-	AA, AGA, CIA, DLE, FD, FFA, LPP, fMF, PsA, LSC	6, 7, 8, 10, 11, 20, 27, 36, 37
Melanophages	Plumped, polygonal to stellate bright cellular structure, larger than inflammatory cells	Melanophages	-	AA, AGA, DLE, FFA, LPP	6, 7, 11, 20
Dermal sclerosis/fibrosis/scarring/dermal fibre degeneration	Increased number of white, ill-defined, coarse dermal fibers at the level of the superficial dermis/dermal fibre degeneration/white papillae	Dermal sclerosis/abnormal collagen bundles/scarring	White structureless areas lacking follicular openings	AA, AGA, CIA, DLE, FD, FFA, LPP, fMF, LSC, ECDS	6, 7, 10, 11, 20, 26, 28, 29, 32, 33, 36, 37, 38, 46
Dilated vessels	Prominent round or linear dark canalicular structures within the dermal papillae/in the upper dermis; in vivo blood flow within these structures is visible	Dilated vessels	Perifollicular erythema/blood vessels	AA, AGA, DLE, FD, FFA, LPP, PsA, LSC	6, 11, 20, 27, 28, 32, 33, 36
Perivascular inflammatory infiltrates	Single, aggregates or diffuse round-to-polygonal, mildly refractive cells located around the vessels	Perivascular inflammatory cells	-	DLE	^28^

Definitions are author-harmonized summaries of published descriptions. The supporting source is given for each feature and no new terminology is introduced. Glossary terms and definitions were extracted from the included full texts by 1 reviewer (J.Ż.), and the completed tables were reviewed, corrected where necessary, and approved by all authors, each of whom has more than 4 years of experience in confocal imaging and in the clinical management of hair disorders.

*AA*, Alopecia areata; *AGA*, androgenetic alopecia; *CIA*, chemotherapy-induced alopecia; *DEJ*, dermo-epidermal junction; *DLE*, discoid lupus erythematosus; *ECDS*, en coup de sabre scleroderma; *FD*, folliculitis decalvans; *FFA*, frontal fibrosing alopecia; *fMF*, folliculotropic mycosis fungoides; *HHPi*, Hedgehog pathway inhibitor; *LPP*, lichen planopilaris; *LSC*, lichen simplex chronicus; *PsA*, psoriatic alopecia; *RCM*, reflectance confocal microscopy.

**Table V:** Glossary of line-field confocal optical coherence tomography features in hair loss

LC-OCT term	Definition	Histopathologic correlate	Trichoscopy correlate	Entities	Source (reference)
Epidermal features					
Exocytosis	Small, single to aggregate bright round cellular structures visible in the epidermis	Exocytosis	-	FFA, LPP	^31^
Pigment incontinence	Epidermal pigment incontinence	Pigment incontinence	-	FFA, LPP	^31^
DEJ-associated features					
Reduced undulation of the DEJ profile	Reduced undulation of the DEJ profile	Flattening of the DEJ	-	AA	^43^
Interface changes	Small, bright, round cellular structures, as singles or tightly aggregated, at the level of the dermo-epidermal junction and/or contiguous hyper-reflective junctional band	Interface changes	-	DLE	30, 42
Basal cell vacuolization	Mildly to low refractile round cellular structures at the DEJ, often associated with a complete annular bright ring with polar enhancement surrounding a dark center corresponding to signet ring-like cells	Keratinocyte necrosis and apoptosis	-	DLE	30, 42
No rimming of the papillae	No visibility of roundish regularly arranged structures at the epidermis–dermis border corresponding to dermal papillae	DEJ damage secondary to interface changes	-	DLE, FFA, LPP	30, 31
Adnexal and periadnexal features					
Yellow dots	Hyper-reflective amorphous material filling a dilated hair infundibulum (filling only upper portion of the infundibulum with a hyporeflective, roundish area below the plug)	Hair follicle filled with keratotic material, with sebum below	Yellow dot surrounded by a thin, bright yellowish collarette (keratin plug)	AA	23, 43
Yellow dots (HHPi-induced alopecia)	Dilated follicular dark spaces containing malted, slightly bright, nonhomogeneous material outlined by brighter collarettes – collection of bright amorphous material along the entire length of the infundibula	-	Yellow dots	HHPi-induced alopecia	^15^
Yellow-white dots	Hyper-reflective amorphous material filling a dilated hair infundibulum (down to mid dermis) with hyper-reflective white strands around follicular ostium	Hair follicle filled with sebum and/or keratotic material; hyperkeratinization occurring at the ostium site of the hair follicle walls	Bright yellow-whitish dot surrounded by a thin brownish collarette	AA	^43^
Black dots	Elongated thin hyporeflective structure, with parallel walls and hyper-reflective spots inside/irregular, hyper-reflective amorphous material filling the infundibulum	Hair medulla remnants, cadaverized hair	Black dots	AA, HHPi-induced alopecia	15, 18, 23, 43
Periadnexal/perifollicular inflammatory infiltrates	Small, bright, round cellular structures tightly aggregated, forming a congruent whitish mass around the hair follicle corresponding to lymphocytes located around hair follicle	Periadnexal inflammatory cells	-	HHPi-induced alopecia, DLE, DC, FD, FFA, LPP	15, 21, 22, 31, 42
Adnexal inflammatory infiltrates∗	Small, single to aggregate bright round cellular structures visible in the adnexal structures	Infiltration of inflammatory cells in adnexal structures	-	AA∗	Present report (Fig 2)
Follicular remnants/plugs	Roundish areas associated with former hair follicles, filled with bright material and necrotic edematous structures	-	Follicular plug	DLE	30, 42
Pustule/abscess	Dense, roundish, hyper-reflective area with well-defined borders filled with multiple hyper-reflective, round cells	Pustule/abscess	Pustule/inflammatory nodule	DC, FD	22, 42
Absence of hair follicles	No visibility of roundish compact structures surrounded by adnexal epithelium corresponding to hair follicles	Absence of hair follicles	No visibility of hair follicles	FFA, LPP	^31^
Destructed hair follicles	Roundish areas associated with former hair follicles, filled with bright material and necrotic edematous structures	-	-	FFA, LPP	^31^
Infundibular hyperkeratosis	Bright material filling the follicular ostia	Infundibular hyperkeratosis	-	FFA, LPP	31, 42
Perifollicular hyperkeratosis/scaling	Bright material filling the ostium of the hair follicle and surrounding the hair shaft	Perifollicular hyperkeratosis	Peripilar casts	FFA∗, LPP	^21^; present report (Fig 3)
Perifollicular fibrosis/scarring	Increased number of white, ill-defined, coarse dermal fibres surrounding the hair follicle	Perifollicular fibrosis/scarring	-	DLE, DC, FD, FFA, LPP	21, 22, 42
Follicular fusion	Coalescence of adjacent follicular units within a common expanded infundibulum	-	-	DC	^42^
Hair shaft-related features					
Broken hairs	Intrainfundibular portion of the hair shaft caliber ranging from 50 to 85 μm and a well-defined, hyper-reflective structure showing a tubular or roundish morphology if visualized in its longitudinal or transverse section, respectively	-	Terminal hairs fractured a few millimeters above the surface level of the scalp skin	AA, HHPi-induced alopecia	15, 23
Exclamation mark hairs/tapered hairs	Intrainfundibular portion of the hair shaft caliber ranging from 15 to 45 μm/short dark dysmorphic hairs with different sizes of proximal and distal ends	-	Exclamation mark hairs/tapered hairs	AA, HHPi-induced alopecia	15, 23
Vellus hairs	Thin, linear, hyper-reflective structures with a caliber ranging from 5 to 10 μm	Vellus hair shafts	Vellus hairs	AA	23, 45
Endothrix type hyphae	Structures filled with small hyper-reflective white dots almost homogeneously distributed and a bright border	Endothrix type hyphae	-	Tinea capitis	^16^
Hair tufts	Multiple hair shafts emerging from dilated hair follicle	Fibrous tissue contraction causing an epidermal depression and clustering of the inflamed follicular units that open into a common ostium	Hair tufts	DC, FD	22, 42
Abnormal hair shaft morphology∗	Abnormal shape and/or degeneration of the hair shaft	-	Pili torti	FFA∗	Present report (Fig 3)
Dermal features					
Dermal sclerosis/abnormal collagen bundles/scarring	Hyper-reflective linear structures in the upper dermis, arranged in different directions, corresponding to thickened collagen bundles/Increased number of bright, ill-defined, coarse dermal fibers	Dermal sclerosis/abnormal collagen bundles/scarring	White structureless areas lacking follicular openings	DLE, DC, FD, FFA, LPP	21, 22, 30, 31, 42
Melanophages	Polygonal bright cellular structures in the upper dermis	Melanophages	-	DLE, FFA, LPP	30, 31
Mucin deposits	Dark, hyporeflective, black ribbon-like structures in the superficial and upper dermis, intermingled with undefined bright areas	Mucin deposits	-	DLE	30, 42
Dilated vessels	Nonreflective dark, wide, elongated areas in the dermis	Dilated vessels	Perifollicular erythema/blood vessels	FD, FFA, LPP, DLE∗	21, 22, 31, 42; present report (Fig 4)
Hypervascularization	Increased number of black horizontally oriented structures in the dermis	Increased number of blood vessels	Perifollicular erythema	FFA, LPP	^31^
Dermal inflammatory infiltrates∗	Small, single to aggregate bright round cellular structures visible in the dermis	Dermal inflammatory cells	-	AA∗, DLE∗, LPP	21, 42; present report (Figs 2 and 4)

Terminology definitions and methodology correspond to Table IV. Features additionally extracted from figures in this report are marked with an asterisk.

*AA*, Alopecia areata; *DC*, dissecting cellulitis; *DEJ*, dermo-epidermal junction; *DLE*, discoid lupus erythematosus; *FD*, folliculitis decalvans; *FFA*, frontal fibrosing alopecia; *HHPi*, Hedgehog pathway inhibitor; *LC-OCT*, line-field confocal optical coherence tomography; *LPP*, lichen planopilaris.

∗ Features illustrated in the present report in entities in which they have not been described in the included LC-OCT studies. These features did not contribute to the synthesis, quantitative analysis, or conclusions.

### Noncicatricial alopecias

In AA, the most frequent RCM features included rimmed dermal papillae (15/19; 79%), dermal fiber degeneration (26/34; 77%), hair shaft miniaturization (24/33; 73%), and yellow dots/empty infundibular ostia (24/35; 69%). Inflammatory infiltrates were additionally identified in the epidermis (11/20; 55%), dermis (14/33; 42%), and adnexal structures (12/34; 35%), whereas melanophages (5/19; 26%) and dilated vessels (3/19; 16%) were infrequent (Fig 5).^9^, ^11^, ^29^, ^37^ In AA incognita, follicles in telogen or catagen and telogen germinal units were identified in almost all evaluated cases.^37^ On LC-OCT, 2 morphologically distinct subtypes corresponding to trichoscopic yellow dots were characterized: hyper-reflective amorphous material filling the infundibulum down to the mid-dermis (bright yellow-whitish dots on trichoscopy) was observed in 63/85 cases (74%), whereas hyper-reflective material overlying a hyporeflective roundish area (yellow dots with a bright collarette) was identified in 44/96 cases (46%). Follicular remnants/black dots were present in 59/96 cases (62%), and reduced undulation of the dermo–epidermal junction (DEJ) profile was reported in all cases in 1 series (20/20; 100%) (Fig 2).^18^, ^23^, ^43^, ^45^

In androgenetic alopecia (AGA), rimmed dermal papillae (29/35; 83%) and hair shaft miniaturization (26/36; 72%) were the most common RCM features. Additional findings included dermal fibrosis (18/35; 51%), bright material filling follicular ostia corresponding to trichoscopic yellow dots (17/35; 49%), epidermal inflammatory infiltrates (11/35; 31%), and periadnexal inflammatory infiltrates (10/30; 33%).^11^, ^34^, ^35^ No LC-OCT studies on AGA were identified.

RCM also identified fungal hyphae as a diagnostic clue in tinea capitis, with ectothrix conidia appearing as round hyper-reflective structures measuring approximately 5 to 10 μm surrounding the proximal hair shaft, and hyphae as elongated hyper-reflective structures external to the hair shaft.^14^, ^16^, ^19^, ^24^, ^44^

Chemotherapy-induced alopecia (CIA) had the largest RCM dataset among noncicatricial entities (3 original studies; 111 patients).^8^, ^17^, ^38^ CIA dots defined as mashed, nonhomogeneous material within dilated hair follicles on RCM and corresponding to a yellow structureless area surrounded by a thin white collarette on trichoscopy were the most characteristic feature (71/87; 82%) (Fig 6). Dystrophic follicular patterns (22/24; 92%), black dots (82/111; 74%), yellow dots corresponding to empty adnexal lumina filled with refractile material (78/111; 70%), hair shaft miniaturization (69/101; 68%), and abnormal hair shaft morphology (64/111; 58%) were also observed. No LC-OCT studies on CIA were identified.

In Hedgehog pathway inhibitor (HHPi)–induced alopecia, RCM revealed CIA-like dots and classic yellow dots similar to those observed in CIA.^12^ On LC-OCT, HHPi-induced alopecia additionally demonstrated dilated follicular dark spaces filled with nonhomogeneous matted material, short dysmorphic exclamation mark hairs, broken hairs, and perifollicular inflammatory cells, suggesting a common imaging phenotype of drug-induced follicular toxicity.^12^, ^15^

### Cicatricial alopecias

In discoid lupus erythematosus (DLE), the most frequent RCM features included epidermal disarray (14/14; 100%), necrotic keratinocytes (12/12; 100%), exocytosis (12/14; 86%), and interface changes (11/15; 73%). Inflammatory infiltrates were additionally identified in the epidermis (12/14; 86%) and adnexal structures (9/15; 60%), consistent with the histopathological pattern of the disease. Dilated vessels, abnormal collagen bundles, hyperkeratotic and dilated infundibula, and spongiosis were also frequently observed.^6^, ^28^ On LC-OCT, interface changes (18/19; 95%), dermal sclerosis (18/19; 95%), absent dermal papilla rimming (14/15; 93%), and mucin deposits (15/17; 88%) were most frequent (Fig 4).^30^, ^42^ A continuous hyper-reflective junctional band with basal cell vacuolization was additionally reported in all cases in 1 series. Mucin deposits were exclusively reported in LC-OCT studies and showed strong histopathological correlation.

Follicular fusion on LC-OCT was reported exclusively in dissecting cellulitis, which additionally demonstrated confluent hyporeflective cavities with scattered hyper-reflective dots corresponding to pustules and abscesses, dense perifollicular inflammatory infiltrates, perifollicular fibrosis, and dermal sclerosis.^42^ In folliculitis decalvans (FD), dilated vessels (25/25; 100%), exocytosis (15/15; 100%), fibrosis (23/25; 92%), obscuration of dermal papillae (23/25; 92%), and hair tufts (30/39; 77%) predominated on RCM.^25^, ^32^, ^33^ Follicular pustules, a hallmark clinical and trichoscopic feature of FD, were identified on RCM as round-to-oval structures with a well-defined dark halo containing reflective amorphous material in 64% of cases (25/39). Perifollicular inflammatory infiltrates (11/15; 73%) and epidermal hyper/parakeratosis (7/14; 50%) were additionally observed. On LC-OCT, hair tufting (8/9; 89%), pustules, dilated vessels, and dense perifollicular inflammatory infiltrates (4/5; 80% each) predominated, with interface changes present in all LC-OCT–evaluated cases.^22^, ^42^ In frontal fibrosing alopecia (FFA) and lichen planopilaris (LPP), LC-OCT findings largely overlapped, reflecting their close clinicopathologic relationship, with dermal sclerosis (FFA 29/29, 100%; LPP 37/38, 97%), perifollicular inflammatory infiltrates, and interface changes predominating (Fig 3). Active FFA lesions were additionally characterized by complete absence of hair follicles (24/24; 100%), follicle destruction (20/24; 83%), and absent dermal papilla rimming (16/24; 67%), with similar scarring features observed in LPP, including absent papilla rimming in 19 of 26 cases (73%) and follicle destruction in 16 of 26 (62%). In the transition zone of both entities, hypervascularization and dilated blood vessels were frequently identified. Infundibular hyperkeratosis was more prevalent in FFA (19/24; 79%) than in LPP (16/27; 59%), representing a potential discriminating feature.^6^, ^7^, ^20^, ^21^, ^31^, ^39^, ^40^, ^42^

Key features useful for differential diagnosis are summarized in Table VI.

**Table VI:** Key reflectance confocal microscopy and line-field confocal optical coherence tomography features useful in differential diagnosis of selected diseases associated with hair loss

Feature	Feature description	Disease
Interface changes	Round-to-polygonal, mildly refractive cells at the DEJ with partial or complete loss of dermal papillae rings; continuous hyper-reflective junctional band	Cicatricial alopecias (DLE, DC∗, FD∗, FFA, LPP)
Necrotic keratinocytes/basal cell vacuolization	Mildly to low-refractile round structures at the DEJ with a bright annular ring and central dark core on LC-OCT, corresponding to pale, ill-defined round-to-polygonal cells larger than keratinocytes on RCM	DLE
Perifollicular hyperkeratosis	Hyperkeratotic, white ring surrounding the hair follicle	FFA, LPP∗
Follicular plugs	Roundish areas associated with former hair follicles, filled with bright material and necrotic edematous structures	DLE∗
Follicular fusion	Coalescence of adjacent follicular units within a common expanded infundibulum	DC∗
Hair tufts	Dilated follicular opening with multiple hair shafts (>5 or 6) emerging from a single opening	DC∗, FD
Pustule	Dome-shaped structure with a black halo and granular hyper-reflective material inside. Interconnecting pustules on LC-OCT may support DC diagnosis	DC∗, FD
Hyphae	Elongated branching hyper-reflective structures with speckled appearance	Tinea capitis, kerion celsi†
CIA dots/CIA-like dots	Mashed, malted, nonhomogeneous material inside the follicular ostia, may contain normal or fragmented hair	Drug-induced hair loss† (CIA, HHPi-induced alopecia)

*CIA*, Chemotherapy-induced alopecia; *DC*, dissecting cellulitis; *DEJ*, dermo-epidermal junction; *DLE*, discoid lupus erythematosus; *FD*, folliculitis decalvans; *FFA*, frontal fibrosing alopecia; *HHPi*, Hedgehog pathway inhibitor; *LC-OCT*, line-field confocal optical coherence tomography; *LPP*, lichen planopilaris; *RCM*, reflectance confocal microscopy.

∗ Reported exclusively in LC-OCT studies.

† Reported exclusively in RCM studies.

Detailed findings for additional entities associated with hair loss^10^, ^13^, ^26^, ^27^, ^36^, ^41^, ^46^ are available via Mendeley at https://data.mendeley.com/datasets/y9m58jsn7d/4.^49^

### Treatment monitoring

Treatment monitoring was reported in 12 studies and clustered around 3 clinical outcomes. First, imaging identified early hair regrowth prior to clinical detection. For instance, LC-OCT revealed vellus hairs within the infundibulum in 4 of 8 patients after 4 weeks of baricitinib, and multiple hairs from a single follicular opening were detected after 4 weeks of upadacitinib despite absent clinical response.^18^, ^45^

Second, the persistence of inflammation or fibrosis helped predict irreversible hair loss or guide treatment escalation. In CIA, persistent periadnexal inflammation predicted permanent hair loss.^38^ In DLE, marked reduction of inflammatory infiltrates on RCM corresponded to clinical remission, while dermal sclerosis persisted.^6^ In LPP, a lack of RCM improvement after 8 weeks prompted treatment escalation and further follow-up showed marked reduction of inflammatory infiltrates, while persistent thickening of radially arranged dermal fibers indicated late-stage fibrotic remodeling.^7^

Third, studies highlighted discordance between microscopic imaging and clinical or trichoscopic assessments. In FD, disease activity assessed on RCM did not correlate with trichoscopic observations,^33^ whereas in AGA, clinical improvement occurred despite persistent miniaturization on RCM.^34^ By contrast, 52-week dupilumab treatment in AA resulted in no significant changes on RCM.^29^

### Pooled prevalence of RCM features

Ten RCM features were reported in both subgroups (Table VII). Seven differed significantly, 6 of which maintained stability across 3 sensitivity analyses, although overall evidence certainty remained very low. Forest plots and Grading of Recommendations, Assessment, Development and Evaluation assessments are available via Mendeley at https://data.mendeley.com/datasets/y9m58jsn7d/4.^49^

**Table VII:** Subgroup differences in pooled prevalence of reflectance confocal microscopy features between cicatricial and noncicatricial alopecias, with GRADE certainty of evidence

RCM feature	Cicatricial alopecias, pooled prevalence (95% CI)	Noncicatricial alopecias, pooled prevalence (95% CI)	*P* value	I^2^ (cicatricial; noncicatricial)	Certainty of evidence (GRADE)	Main reasons for downgrading
Dilated vessels	0.65 (0.43-0.82)	0.22 (0.13-0.35)	<.001	58%; 0%	Very low	Inconsistency; indirectness; publication bias
Exocytosis	0.75 (0.58-0.87)	0.40 (0.26-0.57)	.005	25%; 17%	Very low	Indirectness; publication bias
Adnexal inflammatory infiltrates	0.63 (0.50-0.74)	0.35 (0.23-0.49)	.004	0%; 28%	Very low	Indirectness; imprecision; publication bias
Dermal inflammatory infiltrates	0.62 (0.37-0.82)	0.28 (0.15-0.47)	.032	35%; 63%	Very low	Inconsistency; indirectness; imprecision; publication bias
Melanophages	0.52 (0.22-0.80)	0.20 (0.10-0.36)	.070	50%; 21%	Very low	Inconsistency; indirectness; imprecision; publication bias
Periadnexal sclerosis	0.74 (0.50-0.89)	0.30 (0.19-0.44)	.002	69%; 2%	Very low	Inconsistency; indirectness; publication bias
Rimmed dermal papillae	0.23 (0.05-0.62)	0.86 (0.72-0.94)	.002	82%; 55%	Very low	Inconsistency; indirectness; imprecision; publication bias
Dermal sclerosis	0.73 (0.62-0.81)	0.46 (0.15-0.80)	.174	0%; 90%	Very low	Inconsistency; indirectness; imprecision; publication bias
Yellow dots	0.36 (0.08-0.79)	0.60 (0.39-0.77)	.362	86%; 82%	Very low	Inconsistency; indirectness; imprecision; publication bias
Hair shaft miniaturization	0.22 (0.10-0.39)	0.69 (0.61-0.75)	<.001	N/A; 0%	Very low	Indirectness; imprecision; publication bias

Pooled prevalences are random-effects estimates. *P* values are for the between-subgroup difference. I^2^ is reported for the cicatricial and noncicatricial subgroups, respectively. The certainty of the evidence was assessed using the GRADE approach. The full GRADE assessment, including all domains, is provided in Supplementary File 7, available via Mendeley at https://data.mendeley.com/datasets/y9m58jsn7d/4 (Mendeley Data). Post hoc sensitivity analyses excluding single-patient cohorts, excluding case reports, and restricting the analysis to 1 cohort per publication are reported in Supplementary File 8, available via Mendeley at https://data.mendeley.com/datasets/y9m58jsn7d/4 (Mendeley Data).

*CI*, Confidence interval; *GRADE*, Grading of Recommendations, Assessment, Development and Evaluation; *N/A*, not applicable; *RCM*, reflectance confocal microscopy.

## Discussion

Among noncicatricial alopecias, CIA is supported by the most substantial body of RCM evidence. CIA dots were detected more frequently with RCM than with trichoscopy, and RCM has been used to propose a pathogenetic sequence underlying their development.^38^ Persistent periadnexal inflammation identifies patients at a risk of permanent CIA. CIA-like dots in HHPi-induced alopecia suggest that this feature may represent a general imaging phenotype of drug-induced follicular toxicity rather than being specific to cytotoxic chemotherapy. In AA, LC-OCT may detect subclinical hair regrowth before any clinical or trichoscopic change, supporting more personalized treatment. Evidence in AGA remains limited.

Among cicatricial alopecias, LPP and FFA findings overlap substantially, reflecting their clinicopathological proximity. LC-OCT may add discriminatory value in DLE by visualizing mucin deposition, a relatively specific histopathological clue. Interface alterations at the DEJ, with secondary loss of papillary rimming, are strong clues for cicatricial alopecia regardless of subtype. Fibrosis itself should not be regarded as a definitive marker, since sclerotic changes occur across various alopecia subtypes. Among neutrophilic cicatricial alopecias, follicular fusion was reported only in dissecting cellulitis. In morphea, psoriasis, DLE, or tinea, imaging findings often parallel those on nonhair-bearing skin,^50^, ^51^ allowing cautious extrapolation of extra-scalp criteria.

The pooled analysis revealed differences in multiple RCM features between cicatricial and noncicatricial alopecias. Higher prevalence of exocytosis and adnexal infiltrates in cicatricial forms reflects epidermal and follicular inflammation. Periadnexal sclerosis aligns with fibrotic remodeling responsible for irreversible follicular destruction. Dilated vessels in cicatricial alopecias may reflect chronic vascular remodeling. Conversely, rimmed dermal papillae predominated in noncicatricial alopecias, mirroring preserved DEJ architecture. Hair shaft miniaturization predominated in noncicatricial forms. Due to substantial variation in I^2^ across features and very low certainty of evidence, pooled prevalences should be interpreted as descriptive and exploratory rather than as indicators of diagnostic accuracy or predictive value. In practice, these prevalence patterns may help differentiate cicatricial from noncicatricial alopecia rather than serving as standalone diagnostic criteria.

Reported findings overlapped, but no study evaluated both modalities in the same cohort. Necrotic keratinocytes in DLE were more frequently observed with RCM, whereas deeper interface changes, dermal sclerosis, and mucin deposits were more commonly detected with LC-OCT. LC-OCT additionally offers 3-dimensional reconstruction and real-time trichoscopic integration, whereas RCM requires the VivaScope 1500 for trichoscopic correlation, which is less practical for scalp imaging.

RCM and LC-OCT add diagnostic information beyond trichoscopy by revealing inflammatory infiltrates, DEJ alterations, and dermal remodeling. They may be most useful for treatment monitoring and when biopsy is contraindicated or declined, repeated sampling is impractical, trichoscopy is inconclusive, or the most active site must be localized. However, neither modality currently replaces histopathology and diagnostic-accuracy studies benchmarking them against it are an essential next step.

Limitations include small samples, heterogeneous reporting, and reporting bias from numerous case reports and series, which may inflate feature prevalences. Consecutive recruitment with prespecified imaging targets would reduce this bias. Inconsistent terminology complicates data synthesis. Although a nomenclature glossary was proposed, a Delphi-based consensus would be preferable. No studies focused on skin-of-color populations, an important gap given the potential of noninvasive microscopy where erythema-based clues are less evident. Future studies should recruit phototypically diverse cohorts and stratify feature performance by skin tone. Evidence is particularly limited for LC-OCT, and several RCM-characterized structures remain undescribed. Table VIII summarizes recommendations for future studies.

**Table VIII:** Recommendations for future reflectance confocal microscopy and line-field confocal optical coherence tomography research in hair loss

Element	Recommended reporting
Minimum reporting set	
Clinical and trichoscopic data	Clinical diagnosis and hair-loss type; trichoscopic features, disease duration and prior treatment (method and duration); clinical and trichoscopic stage/activity (stable vs active)∗
RCM/LC-OCT device	RCM: eg, VivaScope (Caliber I.D.) LC-OCT: eg, deepLive (DAMAE Medical)
Imaging mode	RCM: VivaScope model (1500 or 3000) and acquisition mode (VivaStack and/or VivaBlock) LC-OCT: imaging mode (vertical, horizontal, and/or 3D)
Imaging planes and depth	Levels and compartments examined (epidermis, dermo-epidermal junction, infundibulum, isthmus, bulb; follicular, perifollicular, interfollicular compartments), imaging depth, and the total number and type of acquired images
Lesion selection and acquisition protocol	Scalp region/location; active edge vs center/inactive zone; number of sites and number of images per site; consecutive vs selective sampling
Acquisition standardization	Scalp preparation (no recent dyeing) and hair parting for visualization
Standardized feature definitions	Use of a proposed glossary (eg, Tables IV and V)
Correlates	Trichoscopy (mandatory); histopathology when available
Patient descriptors	Age, sex, Fitzpatrick phototype
Assessment method	Number of readers and blinding of feature extraction; inter-reader reliability
Proposed protocol for head-to-head (RCM and LC-OCT) studies	
Population	Consecutively recruited patients with the evaluated alopecia type(s)
Imaging	The same lesions imaged with both RCM and LC-OCT at the identical site (co-registered using corresponding trichoscopy or a defined anatomical landmark), within a short interval (ideally the same day), under standardized acquisition
Feature extraction	Independent evaluation using the proposed glossary (Tables IV and V) and the minimum reporting set (see above), with readers blinded to the comparator modality, histopathology, and the reference diagnosis
Reference standard	Histopathologic correlation from the imaged site when biopsied (evaluated blinded to imaging); for nonbiopsied lesions, a diagnosis based on combined clinical and trichoscopic assessment
End points	Diagnostic accuracy (sensitivity/specificity vs histopathology), inter-reader and intrareader reliability, and head-to-head concordance between modalities
Reporting	Preregistered protocol; reporting of per-feature and per-diagnosis performance
Proposed protocol for treatment monitoring studies	
Imaging schedule	Baseline and standardized follow-up intervals (eg, wk 4, 8, 12, and 24), tracking the same lesion over time
Hair regrowth	Assessment of disease remission/hair regrowth, including early subclinical regrowth, recording the timepoint at which remission or regrowth features first appear
Prediction of irreversible hair loss	Markers of follicular destruction (follicle loss, absent dermal papilla rimming), fibrosis, and persistent inflammation as predictors of irreversible hair loss
Discordance between imaging and trichoscopic or clinical assessment	Imaging findings discordant from trichoscopic or clinical assessment (eg, residual microscopic activity despite clinical or trichoscopic improvement)
Quantitative structural end points	Follicular density (n/mm^2^), hair-shaft diameter, proportion of multi-hair units, and thickness of the epidermis and its individual layers, reported as change versus baseline
Time to imaging improvement	Time to first objective RCM/LC-OCT improvement, compared with time to clinical response
Image archiving	Storage of raw RCM/LC-OCT data
Reporting	(Semi)quantitative scoring of glossary-based features (see above) across all feature domains (inflammatory, fibrotic, vascular, follicular/hair-shaft, dermo-epidermal junction, and others) at each timepoint, interpreted alongside clinical and trichoscopic assessment

*3D*, 3-Dimensional; *LC-OCT*, line-field confocal optical coherence tomography; *RCM*, reflectance confocal microscopy.

∗ Active disease is defined by progressive hair loss, a positive hair pull test, and the presence of concurrent symptoms (eg, pruritus, trichodynia), visible signs of inflammation (eg, erythema, scaling, pustules), or trichoscopic signs of active disease. Clinically stable disease is characterized by the arrest of hair loss progression, a negative pull test, and the absence of clinical symptoms, visible inflammation, or trichoscopic signs of active disease.

## Conclusions

Neither modality replaces clinical examination, trichoscopy, or histopathology, yet each delivers entity-specific clues, including mucin deposition and basal cell vacuolization in DLE, follicular fusion in dissecting cellulitis, CIA-like dots in drug-induced follicular toxicity, and subclinical hair regrowth preceding the clinical response in AA treatment.

## Conflicts of interest

Dr Kurzeja participated in an advisory board (CeraVe Innovation Advisory Board at EADV Paris). Drs Żółkiewicz, Spadafora, Anderson, Nowicki, Pellacani, Rudnicka, and Longo have no conflicts of interest to declare.
